# Supplementary material for: Unaffected fellow eye neovascularization in patients with type 3 neovascularization: Incidence and risk factors
Source: PLoS One. 2021 Jul 19;16(7):e0254186. doi: 10.1371/journal.pone.0254186 (PMC8289018; doi:10.1371/journal.pone.0254186)
Supplement: Attachment — Submitted filename: Response to Reviewers2.docx [file pone.0254186.s003.docx]

Please use the space provided to explain your answers to the questions above. You may also include additional comments for the author, including concerns about dual publication, research ethics, or publication ethics. (Please upload your review as an attachment if it exceeds 20,000 characters)

Reviewer #1: The authors should be congratulated because they address all the reviewers' comments. I have no more issues.

Response) We very much appreciate your comment.

Reviewer #2: I could not find your comments about the following question.
In the Discussion section, the authors did make no mention of the study of PLoS ONE 15(12): e0243458. https://doi.org/10.1371/journal.pone.0243458, which is the latest publication on the topic about onset of RAP lesions (type 3 MNV). This paper must be presented and discussed in the Discussion section of the paper by the authors.

Response) Thank you for pointing this out. The study mentioned above suggested that melanin deficiency in RPE cells is a result of intensive macular stress and that neovascularization may occur in association with several cytokines secreted during this period. Here, intensive macular stress can be induced from ischemic choroidal condition, so our study is thought to show a trend that is not different from the results of the above study. We add this in the discussion section, page 9, line 191-193 and 208-212 in revised manuscript with tract changes.
